# Non-invasive 3D imaging of human melanocytic lesions by combined ultrasound and photoacoustic tomography: a pilot study

**DOI:** 10.1038/s41598-024-53220-y

**Published:** 2024-02-02

**Authors:** Anatoly Fedorov Kukk, Felix Scheling, Rüdiger Panzer, Steffen Emmert, Bernhard Roth

**Affiliations:** 1 Hannover Centre for Optical Technologies, Leibniz University of Hannover, Nienburger Straße 17, 30167 Hannover, Germany; 2 Clinic and Policlinic for Dermatology and Venereology, University Medical Center Rostock, Strempelstraße 13, 18057 Rostock, Germany; 3 Cluster of Excellence PhoenixD (Photonics, Optics and Engineering - Innovation Across Disciplines), Welfengarten 1a, 30167 Hannover, Germany

**Keywords:** Translational research, Biomedical engineering, Skin cancer, Preclinical research

## Abstract

The accurate determination of the size and depth of infiltration is critical to the treatment and excision of melanoma and other skin cancers. However, current techniques, such as skin biopsy and histological examination, pose invasiveness, time-consumption, and have limitations in measuring at the deepest level. Non-invasive imaging techniques like dermoscopy and confocal microscopy also present limitations in accurately capturing contrast and depth information for various skin types and lesion locations. Thus, there is a pressing need for non-invasive devices capable of obtaining high-resolution 3D images of skin lesions. In this study, we introduce a novel device that combines 18 MHz ultrasound and photoacoustic tomography into a single unit, enabling the acquisition of colocalized 3D images of skin lesions. We performed in vivo measurements on 25 suspicious human skin nevi that were promptly excised following measurements. The combined ultrasound/photoacoustic tomography imaging technique exhibited a strong correlation with histological Breslow thickness between 0.2 and 3 mm, achieving a coefficient of determination (R^2^) of 0.93, which is superior to the coefficients from the individual modalities. The results procured in our study underscore the potential of combined ultrasound and photoacoustic tomography as a promising non-invasive 3D imaging approach for evaluating human nevi and other skin lesions. Furthermore, the system allows for integration of other optical modalities such as optical coherence tomography, microscopy, or Raman spectroscopy in future applications.

## Introduction

The incidence and mortality of skin cancer have been increasing over the past decades, especially in regions with predominantly fair-skinned population and high sun exposure. It is the most common type of cancer worldwide, with more than 1.5 million new cases estimated in 2020. At the same time for melanoma, which is the most dangerous and invasive type of skin cancer, an estimated 325,000 new cases were diagnosed globally and 57,000 deaths resulted from the disease^1^.

The stage at which melanoma is diagnosed is a major factor in its prognosis, with a 10-year survival rate of 95% for localized melanoma and less than 10% for metastatic melanoma^2^. Early detection and diagnosis are therefore critical for improving survival rates and reducing associated mortality. Currently, the gold standard for diagnosis is visual examination and biopsy for histopathological analysis if the lesion is deemed suspicious. However, this approach is invasive, costly, and time-consuming, which may result in delayed treatment. Additionally, histopathology analysis only evaluates a couple of slices, which may not accurately represent the lesion’s largest depth and could result in the underestimation of its thickness. This, in turn, could lead to inappropriate tumor staging and clinical procedures. Additionally, preoperative knowledge about size and depth of the lesion would facilitate the planning of the surgery and increase the chance of tumor free excision margins.

As a result, there is a need for alternative fast and non-invasive imaging methods, which would ideally provide the dermatologist with a 3D image of the lesion. Many clinical studies have thus analyzed methods such as optical coherence tomography (OCT)^3^, hyperspectral imaging^4^, reflectance confocal^5,6^, fluorescence^7^, and multiphoton microscopy^8,9^, photoacoustic tomography (PAT)^10,11^, and high-frequency ultrasound imaging (HFUS)^12–14^, or some combination of them^15,16^. Also, photoacoustic microscopy (PAM) has shown successful applications in dermatology and in particular in angiography^17–19^. Table 1 presents a summary of the advantages and disadvantages of various skin imaging modalities for melanoma assessment. Unfortunately, most purely optical imaging methods are often limited in maximal imaging depth and are, therefore, incapable of measuring the depths for all possible ranges to date. On the other hand, ultrasound and photoacoustic tomography enable resolving deeper lesions, although usually at the price of reduced contrast. However, most demonstrated clinical studies are done with B-mode (2D slices) methods and are therefore subject to the same problem as histopathology.

**Table 1:** Summary and comparison of non-invasive modalities for skin imaging and skin cancer assessment.

Modality	Advantages	Limitations
US^12,13,20,21^	- Real-time imaging	- Reported low contrast for lesions^13,21^
	- Cost-effective	
	- Widely available	
PAT^10,11,22^	- Provides functional and structural information	- More complex and expensive than US
	- Good contrast for melanin	- Challenges in clinical implementation
		- Lower resolution than US and PAM
PAM^23,24^	- High spatial resolution	- Long acquisition times
	- Microvasculature visualization	- High exposure
		- Limited penetration depth
OCT^3,14^	- Visualization of epidermal morphology	- Low penetration depth with pigmented lesions
	- High spatial resolution	
	- Real-time imaging	
Raman spectroscopy^25–27^	- Identifies biochemical composition	- Limited to surface area
		- Reported weak signal for exposures under MPE
		- No depth information
Optical microscopy^4,6,8^	- High spatial resolution	- Surface-limited
	- Cellular and tissue structures visualization	

For this study, we have developed a new system that combines ultrasound (US) and photoacoustic tomography (PAT) in a single scanning unit, which is capable of performing a sequence of equidistant 2D slices. This allows to visualize the lesion in 3D, which helps to localize the deepest position and assist the surgeon in the decision of excision margins. The proposed design has a clear optical window above the lesion, which allows it to be combined with other optical modalities in addition. We conducted an in vivo prospective measurement on 25 suspicious human pigmented skin lesions to validate the system for clinical application. The lesions were promptly excised following the imaging measurements for further histopathological analysis. By correlating the obtained imaging data with the corresponding histological findings, we analyzed the accuracy and potential clinical utility of the combined ultrasound and photoacoustic tomography approach in non-invasive 3D imaging of human melanocytic and potentially other skin lesions.

## Materials and methods

### Experimental setup

**Figure 1:**
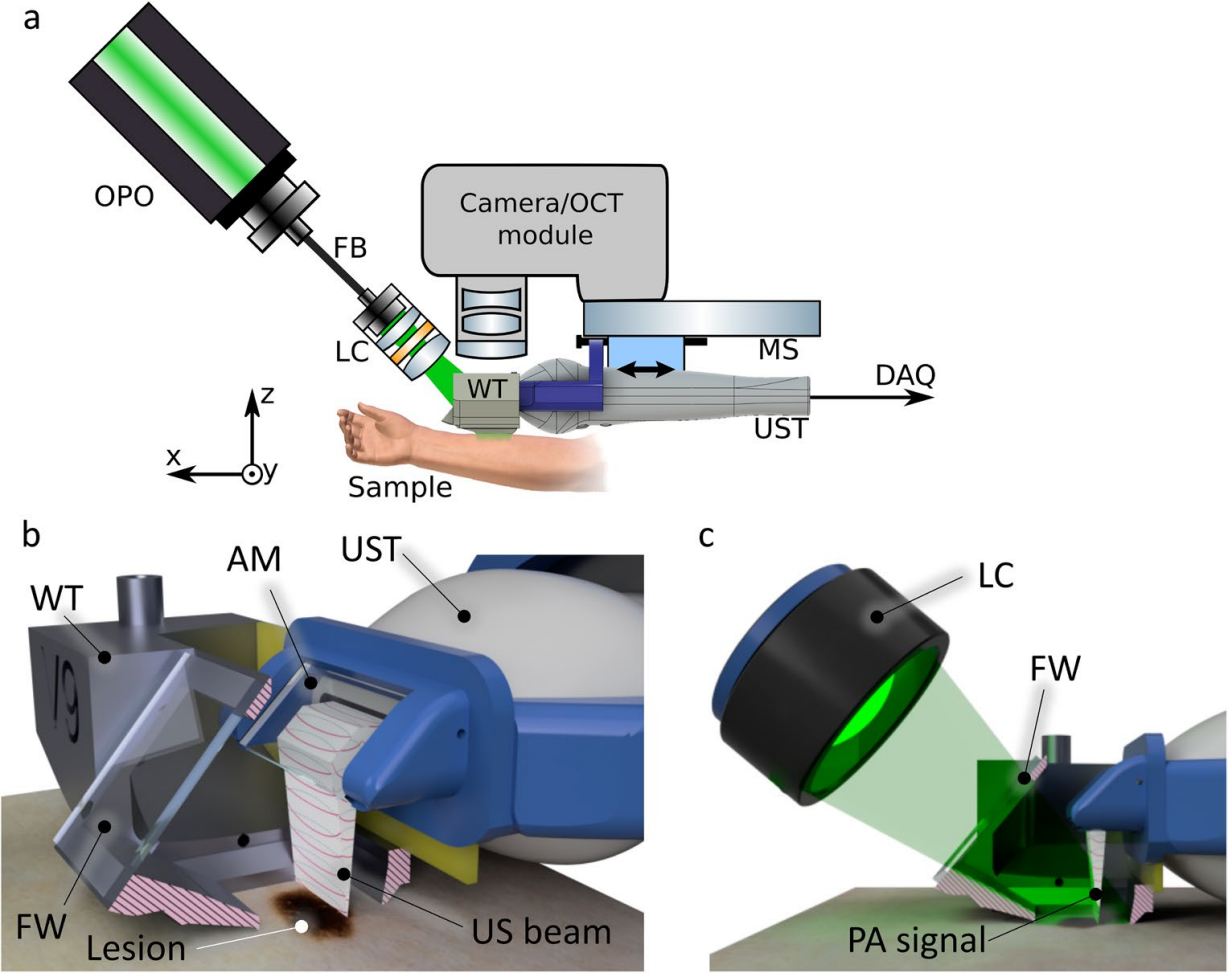
(**a**) Sketch of the experimental setup. The US and PA perform B-mode imaging in the y–z plane; by translating the adapter in x direction with defined steps the 3D imaging is achieved. (**b**) Rendered 3D images of the WT and UST adapter, being positioned on skin. The WT is cut in the middle for a better viewing of the opening and the adapter, revealing how the sketched US path is reflected against the acoustical mirror (AM). (**c**) Illustration of PAT excitation. The laser light is sent though the light collimator (LC) through the front window (FW) of the WT. Abbreviations: *DAQ* data acquisition, *FB* fiber bundle, *MS* motorized stage, *UST* US transducer.

The principle and functionality of the device was presented in our previous work^22^. The experimental setup is represented in Fig. 1. The ultrasound and photoacoustic reception is done with a 18 MHz central single crystal transducer (L22-14vX-LF, Vermon, France) with an acoustical focal length of 20 mm, see^16,22^. It is connected to a research ultrasound system (Vantage 32LE, Verasonics, USA), which generates and acquires the signals at a sampling rate of 62.5 MHz. The transducer is positioned horizontally inside a water tank (WT) that is 3D-printed with resin. The bottom of the WT features an opening of 1 cm x 1 cm, which is placed over the lesion and filled with water as acoustic medium. The transducer adapter consists of a glass slab positioned at a 45-degree angle in front of the detector area. Once the WT is filled, the glass functions as an acoustic mirror (AM) and reflects acoustical waves through total internal reflection, folding the imaging plane onto the WT opening. This allows for optical transparency from the top side (which can be utilized for other optical modalities) and from the front side, which is used for photoacoustic tomography (PAT) illumination. The efficiency of acoustic reflection was measured by comparing the US intensity with and without the AM at the same distance. The negligible difference observed suggests a highly efficient reflection rate (greater than 95%).

**Figure 2:**
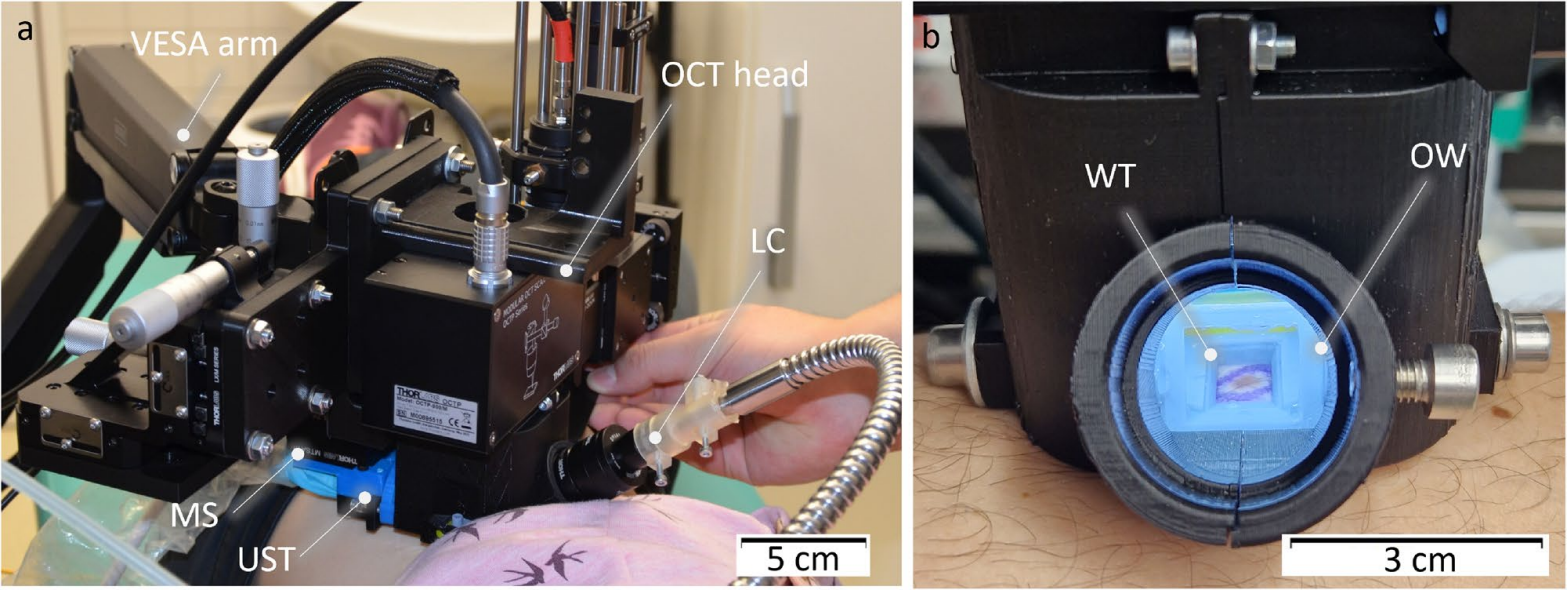
Photograph of the measurement head. (**a**) Photograph of the setup being applied on the patient. Abbreviations: *LC* light collimator, *MS* motorized stage, *OW* optical window, *UST* ultrasound transducer, *WT* water tank. (**b**) Photograph of the WT positioned on skin before each measurement. The skin lesion (brown spot surrounded by blue spindle shape excision marking) can be seen in the tank opening through the frontal optical window that is used for optical excitation (with LC removed). The OCT head was used to integrate the combined US and PA system.

For 3D imaging, the transducer is translated with a motorized stage (MTS25/M-Z8, Thorlabs, USA) within the WT. The scanning head is mounted onto a standard VESA monitor arm, providing support and enabling convenient alignment of the WT aperture with the skin surface. A photograph of the scanning unit is shown in Fig. 2. The optical excitation for PAT is achieved with an optical parametric oscillator (OPO, SpitLight Compact 400 OPO, InnoLas Laser GmbH, Germany), which generates light pulses of 7 ns duration at 20 Hz repetition rate. The pulses are guided via a custom-made optical fiber bundle (CeramOptec GmbH, Germany) and projected through the frontal optical window of the WT with a collimator consisting of 2 achromatic lenses (AC127-019-A and AC254-030-A, Thorlabs, USA) and a square diffuser (ED1-S20-MD, Thorlabs, USA) onto the opening/lesion. Using highly pigmented coffee agar phantoms that mimic melanin absorption, the maximum imaging depth was found to be at least 5 mm. The spatial resolution capabilities of the modalities are summarized in Table S1. The optical density was kept at $\le ~6$ mJ/cm^2^ per pulse, which is below the legal German^28^ and American^29^ Maximum Permissible Exposure (MPE) safety regulations, both of which prescribe 20 mJ/cm^2^ per pulse for excitation in the visible range and at 20 Hz repetition rate. We measured each B-mode with US and 2 PAT modes using optical excitation at 430 nm and 530 nm. Every lesion was measured using a sequence of 36 B-modes with a separation of 250 µm between them, and the acquisition time was less than 50 seconds.

### Image evaluation

The PAT images are beamformed and reconstructed using the open-source MATLAB library MUST^30^, which is based on multiplication of quadrature demodulated signals (IQ) with sparse delay-and-sum (DAS) matrix^31^. For image reconstruction purposes, the speed of sound inside skin tissue was set to 1580 m/s, as suggested by Weichenthal et al.^32^. To account for variations in skin pigmentation, the dynamic range (logarithmic compression factor) for PAT was automatically determined for each lesion using a MATLAB script that analysed and aimed to achieve a specific pixel histogram distribution.

**Figure 3:**
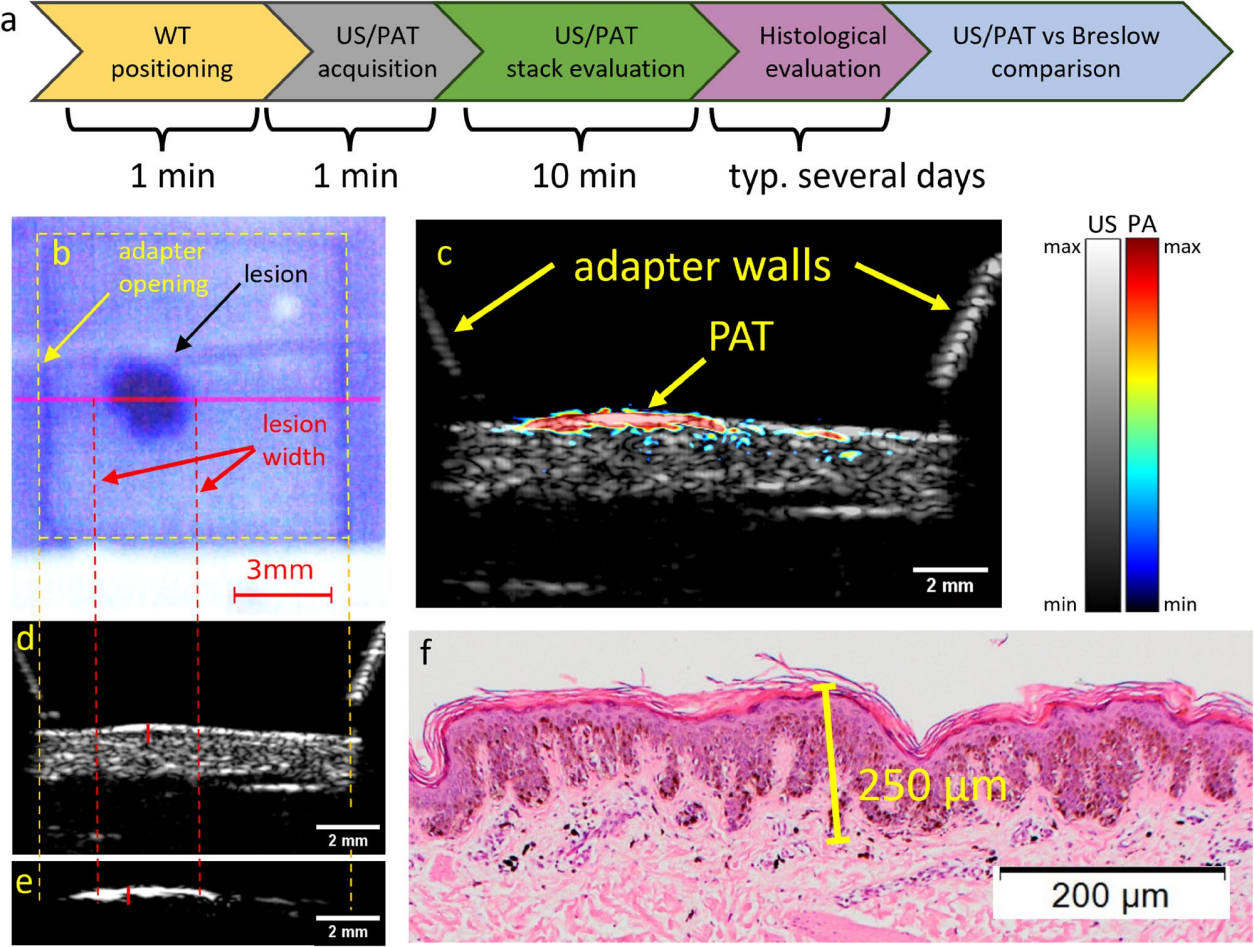
Measurement procedure and example measurement on a lesion (melanocytic junction nevus). (**a**) Block diagram representing the measurement process and the timescale. (**b**) Camera view of the WT opening from the optical window from the top. The pink line across the lesion marks the B-mode shown in (**c**). The prevalence of blue color at the camera image is caused by a 475 nm shortpass filter, which was necessary for additional Raman measurements. The vertical dashed lines mark the WT opening (yellow) and roughly the width of the lesion (red). (**c**) Combined B-mode of the lesion. US is represented in grayscale, PAT in false color. The diagonal lines on top of the skin are the WT walls. (**d**) The US image and the PAT image (**e**) in grayscale. The solid red lines mark the manual measurement of the lesion thickness at its largest depth. (**f**) The corresponding histological measurement, with melanocytic nevus cells at a maximum depth measured at 250 µm, as marked with the yellow line.

The US and PAT images were given to several independent researchers as separate stacks in grayscale. Each researcher independently measured the thickness of the lesion at its thickest position within their respective modality’s stack to localize it, similar to the localization of Breslow thickness by a histologist. Subsequently, the largest thickness identified by each researcher, both in the US and PAT images, was considered as the result for the corresponding modality. An example image of the combined US/PAT measurement, illustrating the details of the localization process, is presented in Fig. 3.

### Clinical setup

The study recruited patients who had suspicious skin lesions identified by dermatologists as potentially indicative of skin cancer. 19 patients (8 female and 11 male) with Caucasian skin type, with ages between 21 and 77 years, with a total number of 25 such skin lesions that were scheduled for excisions participated in the study. Only lesions that were fully measured with the scanning unit are considered for evaluation. From 25 lesions, 4 were only measured with the US, due to a technical problem with the laser operation. The performed measurements were approved by the Ethics Committee of the University Medical Center Rostock (A 2016-0115) and met the principles of the Declaration of Helsinki. All the participants gave written and oral informed consent for the participation in this study. Immediately after the measurements the lesions were excised, fixed with formalin, paraffin-embedded and stained with haematoxylin and eosin(H &E) for histological examination. A dermatopathologist, blinded to the imaging depth results, provided the histological diagnosis and the Breslow’s depth or the equivalent deepest melanocytic infiltration depth for non-melanoma lesions from the histological slices.

## Results and discussion

**Figure 4:**
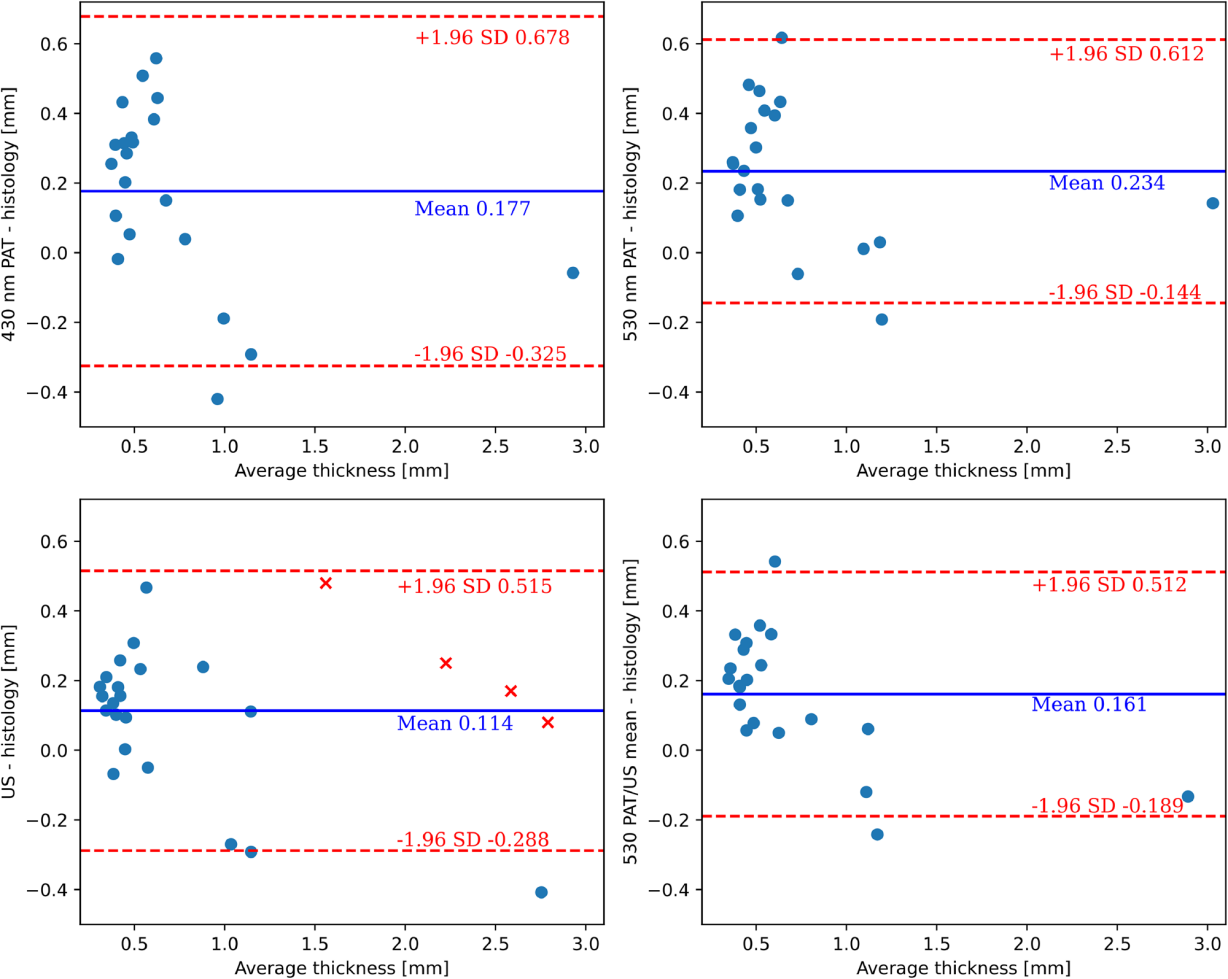
Bland–Altman plots for clinical results. Blue line shows the mean difference between measurement and histology; red lines mark the 95% limits of agreement (± 1.96 standard deviations). (**a**) PAT with radiation at 430 nm and 530 nm (**b**), respectively. (**c**) US and mean of US with PAT 530 nm excitation wavelength (**d**). The red crosses in the US plot represent data points that were not measured additionally with PAT due to a technical problem.

Figure 4 showcases Bland-Altman plots, a widely employed statistical method for gauging the concordance between two measurement techniques. In these plots, the differences between in vivo measurements and histological depth are visualized against their means. The central horizontal line denotes the mean difference, while the upper and lower lines delineate the limits of agreement, signifying the range wherein 95% of the differences are anticipated to reside.

PAT with 430 nm excitation wavelength has the widest spread and tends to overestimate the thickness. It thus shows the largest confidence interval (CI), which may be due to the poor penetration depth of the radiation at this wavelength. On the other hand, 530 nm PAT and US alone lead to better results with a 95% CI of 0.8 mm. On average, all modalities are overestimating the thickness as compared to the Breslow’s depth by 0.1 to 0.2 mm. The most accurate prediction of histological thickness in terms of the mean and CI is found by averaging the results from US and 530 nm PAT, as shown in the bottom right plot of Fig. 4.

On average the overestimation of the thickness compared to the Breslow measurement ranges from 0.1 mm to 0.2 mm. Notably, other clinical studies with non-invasive methods report similar results^12–14,33,34^. Among the modalities, PAT with 430 nm excitation wavelength exhibits the largest inconsistency with the histological values and is therefore the least reliable. US alone can achieve good results and correlates well with histology, which makes it a more feasible and affordable option for clinical practice. However, PAT can further improve the results by enhancing the contrast and specificity of the imaging. The combination of 530 nm PAT and US shows the strongest correlation with Breslow thickness, with an R^2^ of 0.93, as seen in Fig. S2 (supplementary material). Interestingly, these results exhibit a similar pattern of overestimating the thickness for lesions thinner than 0.5 mm and slightly underestimating it for thicker lesions, as reported by other clinical studies using OCT^12,14,33^, 15-25 MHz US^12,13,33,34^ and 100 MHz HFUS^14^. However, at the critical thickness of around 1 mm, which is the cut-off depth to perform sentinel node biopsy, our combined system features a superior performance.

**Figure 5:**
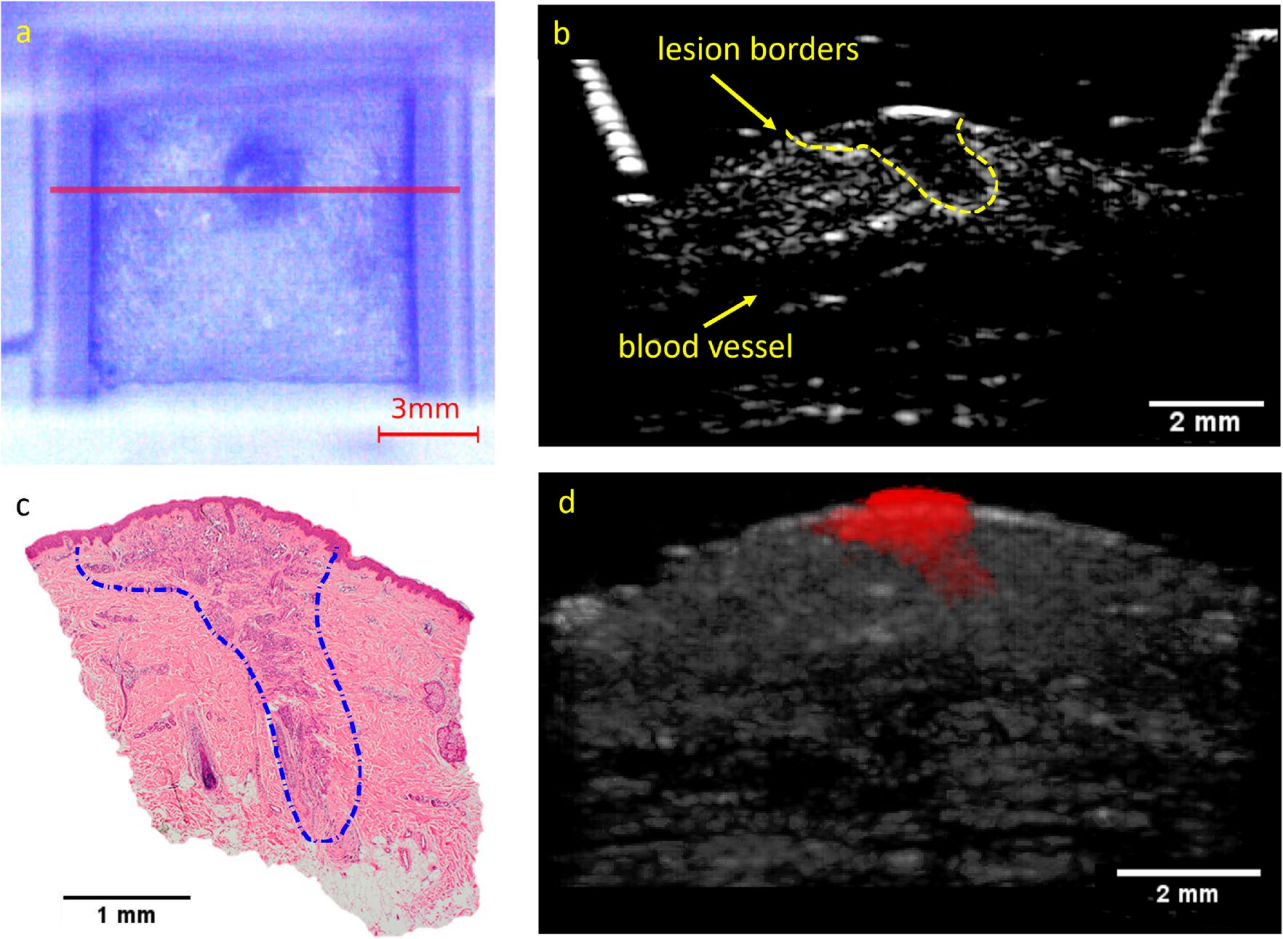
Example of a congenital melanocytic compound nevus with a complex shape. (**a**) Image with camera above, with red line marking the position of the measured B-mode image. (**b**) The corresponding US image, with yellow dash line marking the unusual deep thin shape of the nevus. (**c**) The corresponding histological image, with blue line marking the nevus shape. (**d**) 3D visualization of the lesion (as seen from the x-z plane), with the manually segmented lesion borders denoted by a red color. The volume between the B-mode images was interpolated with tricubic sharp method in ImageJ. A rotation of this lesion in 3D is presented in the supplementary material (Fig. S1).

In comparison to other studies with B-mode HFUS^14,33^ and OCT^14^, which analysed thinner lesions ($\le$ 1 mm) due to the partially limited penetration depth of the OCT or HFUS, our study presents measurement on lesions over a larger thickness range (1–3 mm). Also, other studies that investigated thicker lesions in vivo reported CI in the range >2 mm^12,20,35^, which is significantly above our findings.

In general, the observed overestimation of the modalities as compared to the histological Breslow thickness with regard to thin lesions can be attributed to tissue shrinkage, which occurs immediately after excision and is worsened during the subsequent dehydration and paraffinization stages, resulting in a reduction in histological thickness^36,37^. It is possible that the shrinkage effect is relative to the size, which is less significant for thicker excisions. Additionally, the histology might underestimate the thickness to some degree due to the fact that the excision slice is not necessarily taken at the location where the lesion has its greatest depth, whereas the used modalities provide a set of B-mode images for analysis. Since the predictions of both US and PAT depend directly on the correct value of the speed of sound, which has shown to have variance (as high as 6%) between individual tumors and even between different sites of normal skin^32^, it can contribute to further deviations of in vivo measurements with the histological thickness and the observed slight underestimation for thicker lesions.

Furthermore, the proposed imaging with series of equidistant US/PAT scans is especially useful for evaluating very thin lesions with complex shapes, since it can capture size and depth information in three dimensions, as illustrated in Fig. 5. This is important because thin lesions are often difficult to detect and measure with B-mode imaging modalities. Moreover, complex shapes may introduce errors in the estimation of the lesion thickness, as they may not be parallel to the imaging plane or have irregular boundaries. 3D imaging overcomes these limitations by providing a volumetric representation of the lesion that can be sliced and analysed from different angles and perspectives. This allows for a more accurate and reliable assessment of the lesion morphology and dimensions.

A limitation of this study is the relatively small number of skin lesions examined, due to the challenge of finding and recruiting patients. Another limitation is the water leakage from the 1 cm^2^ opening of the WT, which prevented some measurements from being conducted on certain body parts. This problem is intended to be solved in the following studies by using a different WT with a smaller opening and measurement area. The current measurement accuracy is not limited by fundamental principles, but mostly determined by the equipment used so far. For the PAT modality, the accuracy and the imaging depth can be increased by excitation at longer wavelengths, for example in the range between 600 - 700 nm. Also, an ultrasound transducer with higher central frequency can be used to increase the spatial resolution. Furthermore, angled illumination of the lesion from different sides can be implemented as opposed to illumination from one side as realized in the current setup. Finally, data analysis and image processing based on deep learning algorithms might be able to further increase accuracy^38–40^.

This study paves the way for several avenues of future research and development. Firstly, further clinical trials involving a larger cohort of patients with various skin types and skin lesions will be carried out to validate the performance and reliability of the combined US and PAT system. This would provide additional evidence of its effectiveness in accurately assessing lesion size and depth, ultimately aiding in clinical decision-making. Moreover, the clear optical window incorporated in the proposed design enables integration of optical modalities such as OCT and Raman spectroscopy^41^. OCT can provide cross-sectional images of the skin with high resolution, allowing for detailed visualization of tissue structures and potentially aiding in the differentiation of benign and malignant lesions^42,43^. Raman spectroscopy, on the other hand, can provide molecular information about the lesion, offering insights into its biological composition and assisting in determining its malignancy^25–27,44^. Deep learning-based approaches, such as convolutional neural networks (CNNs), have shown promising results in both classification^45^ and medical image segmentation tasks^43,46^. By training a CNN on annotated 3D images, it is possible to develop robust and accurate segmentation models that can automatically delineate the boundaries of skin lesions. This would not only save time and effort for dermatologists but also provide more consistent and objective measurements for evaluating excision margins. Additionally, with the segmentation of the lesion, a 3D representation could directly be displayed to the dermatologist after scanning the lesion for the entire skin area. Overall, the integration of additional optical modalities, the expansion of clinical trials, and the development of automated segmentation techniques hold promise for enhancing the capabilities and clinical utility of the combined US and PAT system in the future.

## Conclusion

To summarize, we show that the developed combined measurement system can successfully determine the depth of skin lesions in vivo, while being under regulated MPE levels and with acquisition time of <1 min. The results demonstrate that the method can both accurately measure the size and the depth of skin lesions. We obtained a high level of agreement (R^2^ of 0.93, CI of 0.7 mm) between US and PAT, the latter with excitation at 530 nm wavelength, and histopathological measurements, indicating a reliable method for assessing lesion thickness and size before excision. The results are comparable to or better than existing studies with OCT or high frequency ultrasound (HFUS). The approach has the advantage of being non-invasive, fast, and relatively cost-effective, making it suitable for routine clinical use. The combination of the results from the two modalities notably increases accuracy further and allows for improved assessment of infiltration depth and pathophysiology of suspicious lesions, particularly when combined with Raman spectroscopy which is molecular-specific. Moreover, the method can potentially reduce the number of unnecessary biopsies and improve the accuracy of surgical excision planning. Future work will focus on increasing the number of lesions examined and combining the US/PA system with other optical modalities for application in a clinical setting, as well as analyzing the PAT imaging with further laser wavelengths.

### Supplementary Information

Supplementary Information.

## Data Availability

The datasets generated during and/or analysed during the current study are available from the corresponding author on reasonable request.
