# Supplementary material for: Non-invasive 3D imaging of human melanocytic lesions by combined ultrasound and photoacoustic tomography: a pilot study
Source: Sci Rep. 2024 Feb 2;14:2768. doi: 10.1038/s41598-024-53220-y (PMC10837440; doi:10.1038/s41598-024-53220-y)
Supplement: Supplementary file 1 — Supplementary Information. [file 41598_2024_53220_MOESM1_ESM.docx]

**Supplementary material**


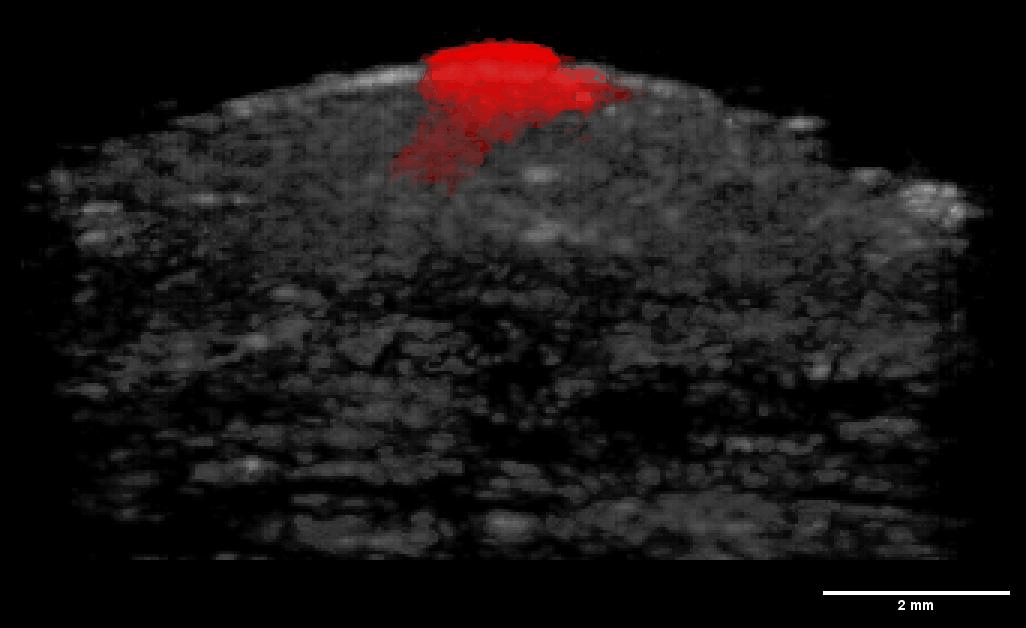


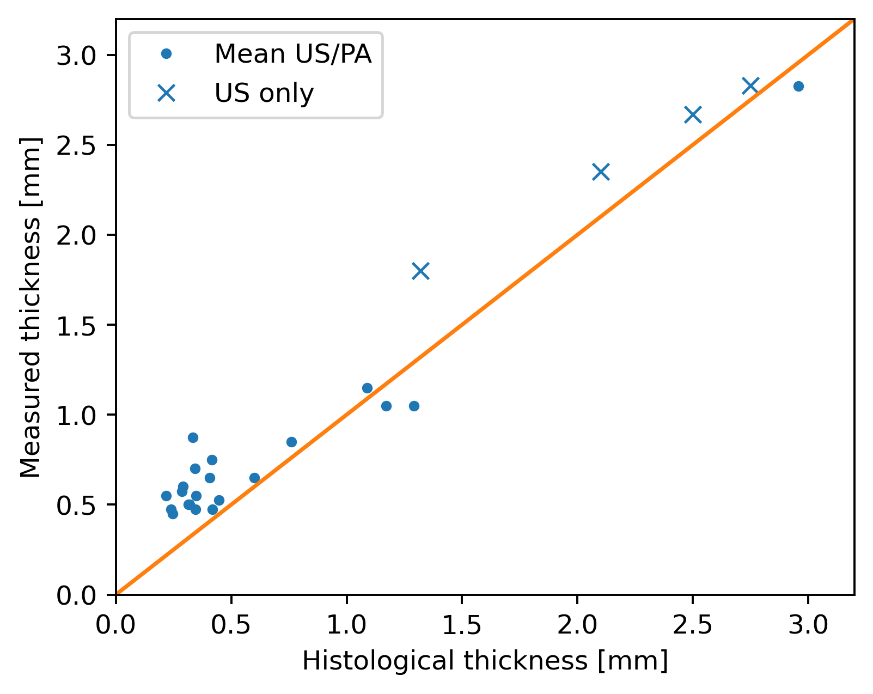
**Figure S1.** Rotation of the volumetric representation (C-mode) of the lesion with an unusual melanocytic invasion shape, which is detailed in Figure 6. The gray area shows the US intensity and the red area marks the manually segmented lesion (by combining the US/PAT intensities). The lesion is classified as congenital melanocytic compound nevus.

**Figure S2.** Average of US with PAT (530 nm excitation wavelength) compared to the histological result. The crosses mark the 4 measurements that were performed only with US due to a technical problem with the OPO. The orange line represents the 1:1 agreement line.

|  | US | PAT (530 nm excitation wavelength) |
| --- | --- | --- |
| Axial resolution (FWHM) | < 125 µm | < 250 µm |
| Axial resolution (Sparrow’s criterion) | < 150 µm | < 280 µm |
| Lateral resolution (FWHM) | < 200 µm | < 300 µm |
| Lateral resolution (Sparrow’s criterion) | < 150 µm | < 350 µm |
| Maximal depth (measured with the agar coffee stairs phantom) | > 5 mm | > 5 mm |

**Table S1.**  Summary of spatial resolution and the maximal depth reachable with the system, as measured with agar phantoms. Note: Sparrow’s criterion was defined as the smallest separation at which 2 strains can be distinguished at different depths inside the cube phantom. The detailed explanation is given in: Fedorov Kukk, Anatoly, et al. "Combined ultrasound and photoacoustic C-mode imaging system for skin lesion assessment." Scientific Reports 13.1 (2023): 17947.
